# Photochromic Polyamide 6 Based on Spiropyran Synthesized via Hydrolyzed Ring-Opening Polymerization

**DOI:** 10.3390/polym13152496

**Published:** 2021-07-28

**Authors:** Shiyou Tian, Jicong Zhang, Qiong Zhou, Limei Shi, Wenwen Wang, Dong Wang

**Affiliations:** 1 Key Laboratory of Textile Fiber and Products (Wuhan Textile University), Ministry of Education, Wuhan 430200, China; 2 Hubei International Scientific and Technological Cooperation Base of Intelligent Textile Materials & Application, Wuhan Textile University, Wuhan 430200, China; 1321274780@163.com (S.T.); zjc1953294170@163.com (J.Z.); wangdon08@126.com (D.W.); 3 SINOPEC Yizheng Chemical Fiber Co., Ltd., Jiangsu Key Laboratory of Highperformance Fiber, Yizheng 211900, China; zhouqiong.yzhx@sinopec.com (Q.Z.); slm1970@126.com (L.S.)

**Keywords:** photochromic, polyamide 6, spiropyran, structure and properties

## Abstract

We report photochromic polyamide 6 (PA6) which was synthesized by hydrolyzed ring-opening polymerization of ε-caprolactam with spiropyran (SP) embedded in the polymer chains. It indicated that crystallinity degree of the resulting copolymers was decreased since only PA6 segments can crystallize with increasing content of SP modifier. Meanwhile, toughness of photochromic PA6 was decreased. The photochromic property analysis indicated that the sample with more flexibility and more content of SP was more sensitive to UV light at the beginning of irradiation than other samples and its color after being irradiated for 1 min tended to reddish. Investigation revealed that the UV-vis absorbance of SP-PA6-3 had negligible decay after 10 cycles, which indicated SP-modified PA6 possessed excellent photoresponse reversibility and fatigue resistance.

## 1. Introduction

The research on preparation and application of photochromic materials has attracted extensive attention due to their obvious and rapid color change, being widely applied in optical information storage [1], chemical sensors [2,3], molecular switches [4], information security encryption [5] and smart textiles [6], etc. Accordingly, various photoswitchable molecules-azobenzenes [7], spiropyrans [8], diarylethenes [9], fulgides [10] and others [11,12] have been widely investigated and employed for the construction of light-responsive systems and materials. As a consequence, spiropyran (SP) is far more than just a simple photoswitch; the range of stimuli able to induce its reversible isomerization is truly impressive and includes different solvents, metal ions, acids and bases, temperature, redox potential and mechanical force [13,14,15,16].

Since last decades, incorporation of photochromic SP into the polymeric matrix could be accomplished by chemical binding via different polymerization reactions and modification of polymer substrate [17,18]. Chemical modification and covalent bonding of SP to the polymer matrix via polymerization will enhance the photostability and photofatigue and the spiropyran moiety can be used in most polymerization conditions. Polymer materials with SP could be found in films [19,20,21], gels [22,23,24] and electrospun nanofibers [25,26,27]. For instance, Mahdavian and coworkers [26] synthesized photoresponsive polymethyl methacrylate and poly(methyl methacrylate-*co*-butyl acrylate) chemically modified with spiropyran through emulsion polymerization and the corresponding nanofibers had excellent responsivity of acid-base vapors. Wu and coworkers [25] developed smart photoresponsive acrylated spiropyran (SPA)-methyl methacrylate (MMA) copolymer films. In addition, spiropyran was covalently attached onto polyamide composite nanofiltration (NF) membranes in a one-step reaction using low-energy electron beam technology and the modified NF membrane showed high removal efficiency of MgSO_4_ when spiropyran transformed into zwitterionic merocyanine [28].

Polyamide 6 (PA6) is a semi-crystalline polymer. High strength and toughness of the polymer are not only provided by the crystallinity but also by hydrogen bonds (H bonds) that form between molecular chains [29]. It is broadly applied as fiber, textile and engineering plastic materials. In textile field, photochromic polyamide materials such as PA fibers or PA fabric are acquired by dyeing or coating with photochromic dyes on their surfaces [6,30]. The photoresponse fatigue resistance and cyclic usage performance need to be improved. Furthermore, it can be seen from the previous work that it is easy to obtain photochromic flexible and amorphous polymers. However, the report for embedding SP into crystalline polymers such as polyamide is rare so far. There are also many influence factors for developing photochromic crystalline polymers, such as chain flexibility, crystallinity degree, molecular weight and SP content, etc.

In this study, we aim to design the chemical structure of PA6 to obtain photochromic PA6 with SP as molecular component to improve the photoresponse fatigue resistance and cyclic usage performance compared to other photochromic PA6 materials with dyeing or coating. Therefore, spiropyran diol was synthesized and then it reacted with adipic acid to obtain the modifier (HOOC-SP-COOH). Amido-terminated polyamide 6 was synthesized by hydrolyzed ring-opening polymerization of *ε*-caprolactam and hexamethylenediamine and then it was modified by HOOC-SP-COOH to get the photochromic PA6. The chemical structures, thermal properties and mechanical properties of photochromic PA6 were characterized. Effect of SP modifier content on photochromic properties of polymers was investigated. The color (RGB) was analyzed and it was displayed in CIE 1931 chromaticity diagram.

## 2. Experimental Section

### 2.1. Materials

ε-Caprolactam (CPL, purity ≥ 99%), adipic acid (purity ≥ 99%), phosphoric acid (H_3_PO_4_, purity ≥ 98%), formic acid (purity ≥ 88%) and methanol (analytical reagent) were purchased from Sinopharm (Beijing, China). *N*,*N*’-dicyclohexyl carbon diimine (DCC, purity ≥ 99%) was purchased from Macklin (Shanghai, China). 4-Dimethylaminopyridine (DMAP, purity ≥ 99%), 1, 6-hexamethylendiamine (HDA, purity ≥ 99%) were purchased from Aladdin (Shanghai, China).

### 2.2. Synthesis of Carboxyl-Terminated Spiropyran (HOOC-SP-COOH)

**Scheme 1:**
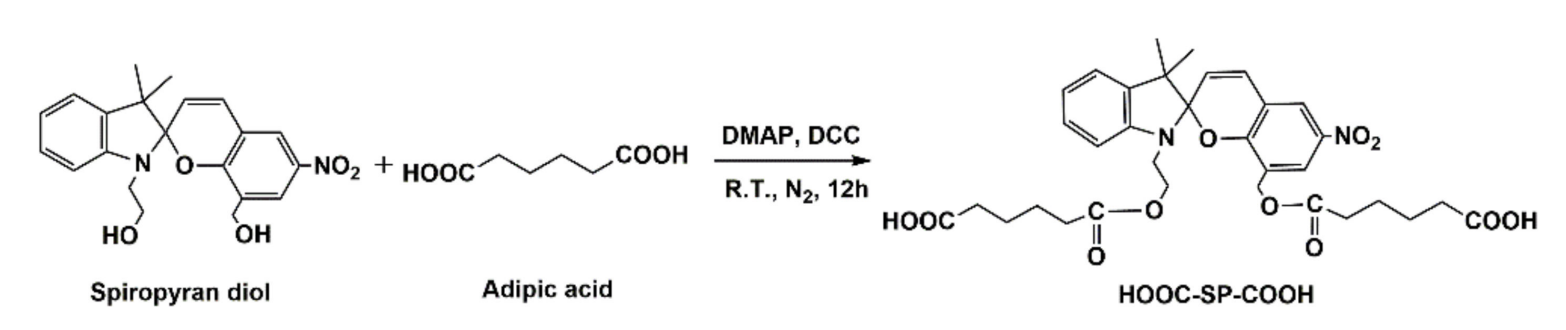
Synthetic route for carboxyl-terminated spiropyran.

Spiropyran diol (HO-SP-OH) was synthesized firstly according to the previous publications [31]. Then, spiropyran diol and adipic acid were used as raw materials to synthesize carboxy-terminated spiropyran derivatives by esterification. The synthetic route is shown in Scheme 1 and the synthetic formulation is shown in Table 1.

The specific synthesis process of HOOC-SP-COOH for SP-PA6-1 is as follows: Spiropyran diol (25.0 mg, 0.065 mmol), excess adipic acid (47.45 mg, 0.325 mmol) and a certain amount of *N*, *N*-dimethylformamide (DMF, 1 mL) were placed into the reaction flask. Appropriate amount of 4-dimethylaminopyridine (DMAP, 7.9 mg) and dicyclohexylcarbodiimide (DCC, 26.9 mg) were added into the reaction flask and the esterification reaction started under stirring at room temperature. Twelve hours later, the product was purified for three times using DMF as solvent and *n*-hexane as precipitant to obtain a spiropyran derivative (HOOC-SP-COOH). The yield of HOOC-SP-COOH for SP-PA6-1 was 80% and the amount of HOOC-SP-COOH was 33.18 mg. The yield of HOOC-SP-COOH for SP-PA6-2 and SP-PA6-3 was 81% (40.58 mg) and 80% (53.45 mg), respectively. The chemical structure of HOOC-SP-COOH was characterized by FTIR, ^1^H NMR and LC-MS. It can be indicated that HOOC-SP-COOH was successfully synthesized according to the above process. The melting point of HOOC-SP-COOH was also measured, which was 182 °C.

### 2.3. Synthesis of Photochromic PA6

**Scheme 2:**
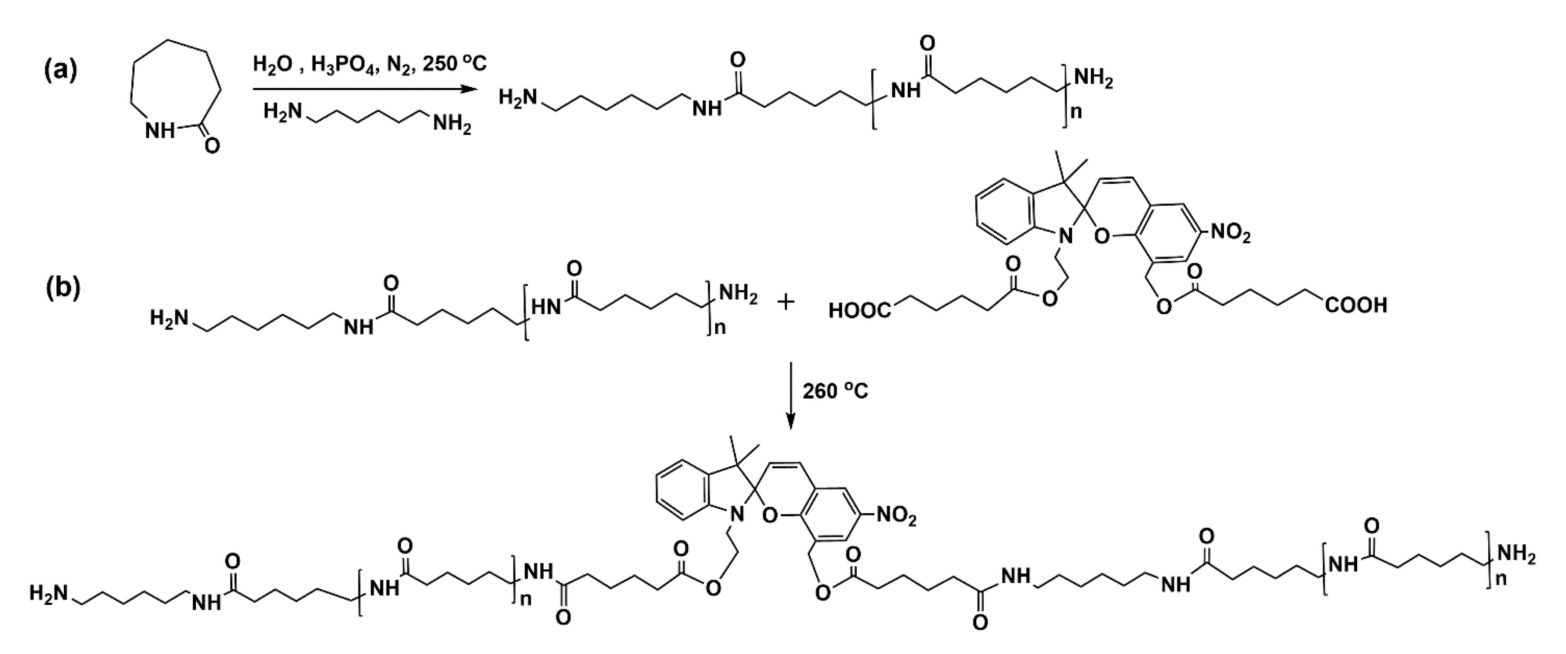
Synthetic route for SP-modified PA6.

The detailed synthesis formulation of photochromic PA6 is shown in Table 1. The typical experimental steps for SP-PA6-1 are as follows: *ε*-Caprolactam (50.002 g), deionized H_2_O (0.501 g), H_3_PO_4_ (1.002 g) and 1, 6-hexamethylendiamine (0.25 g) were added into reaction flask in a flow of nitrogen. The flask was then heated to 250 °C and the ring-opening reaction started under the stirring speed of 100 r/min. Three hours later, the reaction temperature was raised to 260 °C with the stirring speed of 250 r/min. Then, a certain amount of carboxy-terminated spiropyran derivative (HOOC-SP-COOH, 33.18 mg) obtained from the former synthesis step was quickly added to the polymerization system and the polymerization system was pumped on a vacuum line. About 10 min later, the polymerization was terminated and the product was obtained for use. The synthetic route for SP-modified PA6 is shown in Scheme 2. The amount of HOOC-SP-COOH for SP-PA6-2 and SP-PA6-3 was 40.58 mg and 53.45 mg, respectively. The pure PA6 was synthesized just without 1, 6-hexamethylendiamine and carboxy-terminated spiropyran derivative (HOOC-SP-COOH) being added. The polyamides for chemical structure analysis were purified by dissolution–precipitation cycles for three times with formic acid as solvent and methanol as precipitant and then dried in vacuum oven at 100 °C overnight.

## 3. Characterization Methods

### 3.1. Fourier Transform Infrared Spectroscopy (FTIR) 

The chemical structure of HOOC-SP-COOH was investigated by Fourier Transform Infrared Spectroscopy (Tensor 27, Bruker, Ettlingen, Germany).

### 3.2. ^1^H Nuclear Magnetic Resonance Spectroscopy (^1^H NMR) 

The sample was dissolved in deuterated dimethyl sulfoxide (DMSO-d_6,_ Aladdin, Shanghai, China). ^1^H NMR spectrum of HOOC-SP-COOH was obtained on Bruker 400M instrument (Bruker, Ettlingen, Germany) at 23 °C and the number of scans was 48.

### 3.3. Liquid Chromatograph-Mass Spectrometry (LC-MS) 

HOOC-SP-COOH was dissolved in methanol to obtain the solution with a concentration of 10 mg/mL. Liquid chromatography-mass spectrometry (LCMS-2020, Shimadzu, Kyoto, Japan) was used to detect the solution. The mixture of formic acid and acetonitrile is used as the eluent and the sample is ionized by electrospray ionization (ESI, Shimadzu, Kyoto, Japan).

### 3.4. Melting Point

The melting point of spiropyran derivative (HOOC-SP-COOH) was measured by the melting point instrument (YG252A1, Changzhou Shuanggudunda Electromechanical Technology Co., Ltd., Changzhou, China). The preset temperature was 200 °C and the heating rate was 3 °C/min.

### 3.5. ^13^C Nuclear Magnetic Resonance Spectroscopy (^13^C NMR) 

Solid ^13^C NMR spectra of polymers were obtained on Brucker 400M instrument at 25 °C and the number of scans was 572.

### 3.6. Differential Scanning Calorimetry (DSC) 

NETZSCH DSC 204F1 (Netzsch, Selb, Germany) was used to evaluate thermal transitions of polymers under nitrogen purge. The samples were heated from 20 °C to 300 °C at heating rate of 10 °C/min for eliminating thermal history. Then, the samples were heated from 20 °C to 300 °C with heating rate of 10 °C/min and the cooling rate is 20 °C/min for melting peak and crystallization. Crystallization and melting temperature were reported as the temperature at the midpoint of the heat capacity using the software (Netzsch Proteus, Netzsch, Selb, Germany).

### 3.7. Thermogravimetric Analysis (TGA) 

Thermal stability of polymers was examined using TGA instrument (NETZSCH 209 F3 Tarsus, Netzsch, Selb, Germany). Briefly, 5–10 mg of the sample was placed on a platinum pan and then equilibrated at a temperature of 30 °C. The temperature was then raised to 800 °C at 10 °C/min. All TGA tests were performed under a nitrogen atmosphere.

### 3.8. X-ray Diffraction (XRD) 

The crystal phase curves of polymer samples were measured on Empyrean (Panalytical, Malvern Panalytical Inc., Westborough, MA, USA) in the range of 10° to 80°.

### 3.9. Mechanical Properties

The polymers were cut as dog-bone shape with a size of 50 mm × 10 mm and measured at a tensile rate of 20 mm/min on Instron 5965 at room temperature. Tensile toughness (τ) of the samples can be defined by integrating the area under the engineering stress (𝜎)-strain (𝜀) curves, measured at the stretching speed of 20 mm/min, using the following equation:

(1) $$\tau =\int_{\varepsilon =0}^{\varepsilon =\varepsilon_{max}}\sigma d\varepsilon$$

where σ is the engineering stress, ε is the engineering strain, ε_𝑚𝑎𝑥_ is the elongation-at-break of the sample. The error bars of toughness were calculated according to three replicates.

### 3.10. Fluorescence Spectrophotometer 

The samples were prepared into a formic acid solution by using formic acid as solvent with a concentration of 0.03 g/mL and the fluorescence emission spectra of different samples were determined by using Lengguang Tech. F97Pro (Shanghai Lengguang Technology Co., Ltd., Shanghai, China) with excitation wavelength of 523 nm.

### 3.11. UV-Visible Spectrophotometer 

HOOC-SP-COOH was dissolved in ethanol to obtain HOOC-SP-COOH/ethanol solution (0.001 g/mL). The SP-modified PA6 samples were dissolved in formic acid and formic acid solutions with a concentration of 0.03 g/mL were prepared. The UV-Vis spectra of formic acid solutions from different samples and HOOC-SP-COOH/ethanol solution was determined by SHIMADZU UV-2700 analyzer (Shimadzu, Kyoto, Japan).

### 3.12. RGB Analysis 

The RGB value in sample color was analyzed by ImageJ software (V1.8.0.112, National Institutes of Health, Bethesda, MD, USA). The trend of the sample color is then plotted in the CIE 1931 chromaticity diagram.

### 3.13. UV-Vis-Near Infrared Spectrometer 

The sample SP-PA6-3 was irradiated for different time under UV lamp (λ = 365 nm). The absorption was tested using Shimadzu SolidSpec-3700 (Shimadzu, Kyoto, Japan).

### 3.14. Gel Penetration Chromatography (GPC) 

The molecular weight and polydispersity index of PA6 samples were measured by GPC instrument (Agilent PL-GPC50, Agilent Technologies Inc., Palo Alto, CA, USA). A calibration curve was obtained using linear polystyrene (Agilent Technologies Inc., Palo Alto, CA, USA) as a standard and chromatographic grade hexafluoroisopropanol (Sigma-Aldrich, St. Louis, MO, USA) as the mobile phase at an elution rate of 1 mL/min (37 °C). The curves were normalized.

## 4. Results and Discussion

### 4.1. Characterization of Carboxyl-Terminated Spiropyran (HOOC-SP-COOH)

**Figure 1:**
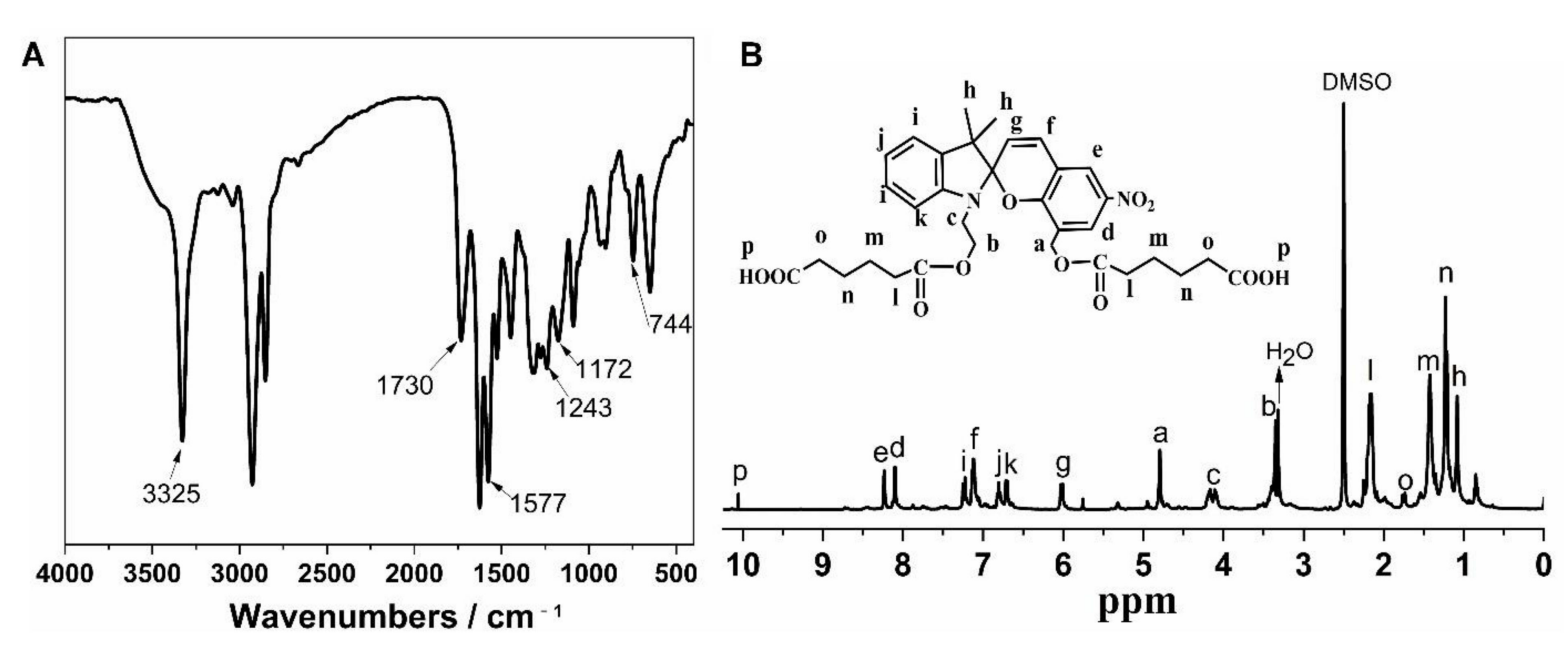
(**A**) FTIR spectrum and (**B**) ^1^H NMR spectrum of carboxyl-terminated spiropyran (HOOC-SP-COOH).

FTIR spectrum of HOOC-SP-COOH can be seen in Figure 1A. Peak at 3325 cm^−1^ is corresponding to the stretching vibration of hydroxyl group. Peaks at 744 cm^−1^ and 1577 cm^−1^ are assigned to the characteristic absorption of benzyl groups in SP structure [32]. The peak at 1172 cm^−1^ is the stretching vibration of C-O-C bond in ester group and 1730 cm^−1^ is the absorption peak of C=O bond in carboxyl group [33]. The peak at 1243 cm^−1^ is assigned to the characteristic absorption of =C-O-C- in SP. Figure 1B displays ^1^H NMR spectrum of HOOC-SP-COOH. The chemical shifts of spiropyran structure can be seen in the spectrum and the peaks are as below: 8.23 ppm (H_e_), 8.09 ppm (H_d_), 7.25 ppm (H_i_), 7.11 ppm (H_f_), 6.81 ppm (H_j_), 6.69 ppm (H_k_), 6.01 ppm (H_g_), 4.78 ppm (H_a_), 4.15 ppm (H_c_), 3.35 ppm (H_b_) and 1.08 ppm (H_h_) [34]. Additionally, the ^1^H NMR spectrum shows two signals at 2.15 ppm, 1.74 ppm, 1.41 ppm and 1.22 ppm corresponding to methyl groups -CH_2_- and a peak associated with the proton of the -COOH group at 10.1 ppm [35]. Moreover, LC-MS was used to analyze HOOC-SP-COOH further and the result can be seen in Figure S1. It shows the high purity of SP derivative.

### 4.2. Structural Characterization of Polymers

**Figure 2:**
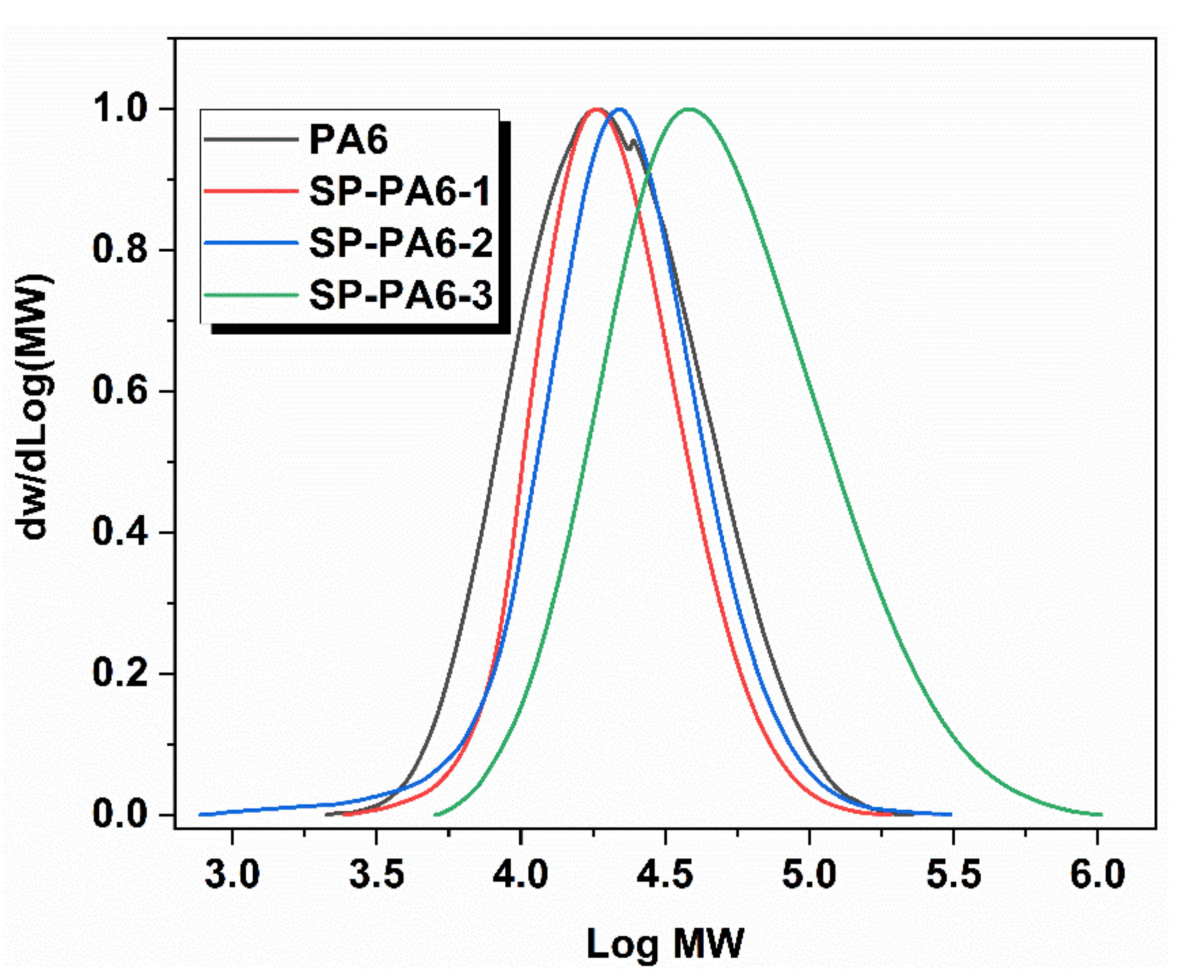
GPC curves of pure PA6 and photochromic PA6.

The photochromic PA6 was synthesized via hydrolyzed ring-opening polymerization using spiropyran derivative as modifier. The characteristic results and GPC curves of samples are shown in Table 2 and Figure 2. It displays that the molecular weight of SP-modified PA6 were increased and higher than that of pure PA6. It is attributed to that SP acted as a linker of PA6 chains. In addition, the molecular weight of SP-PA6-3 was increased a lot because of more HOOC-SP-COOH being added into the polymerization system.

**Figure 3:**
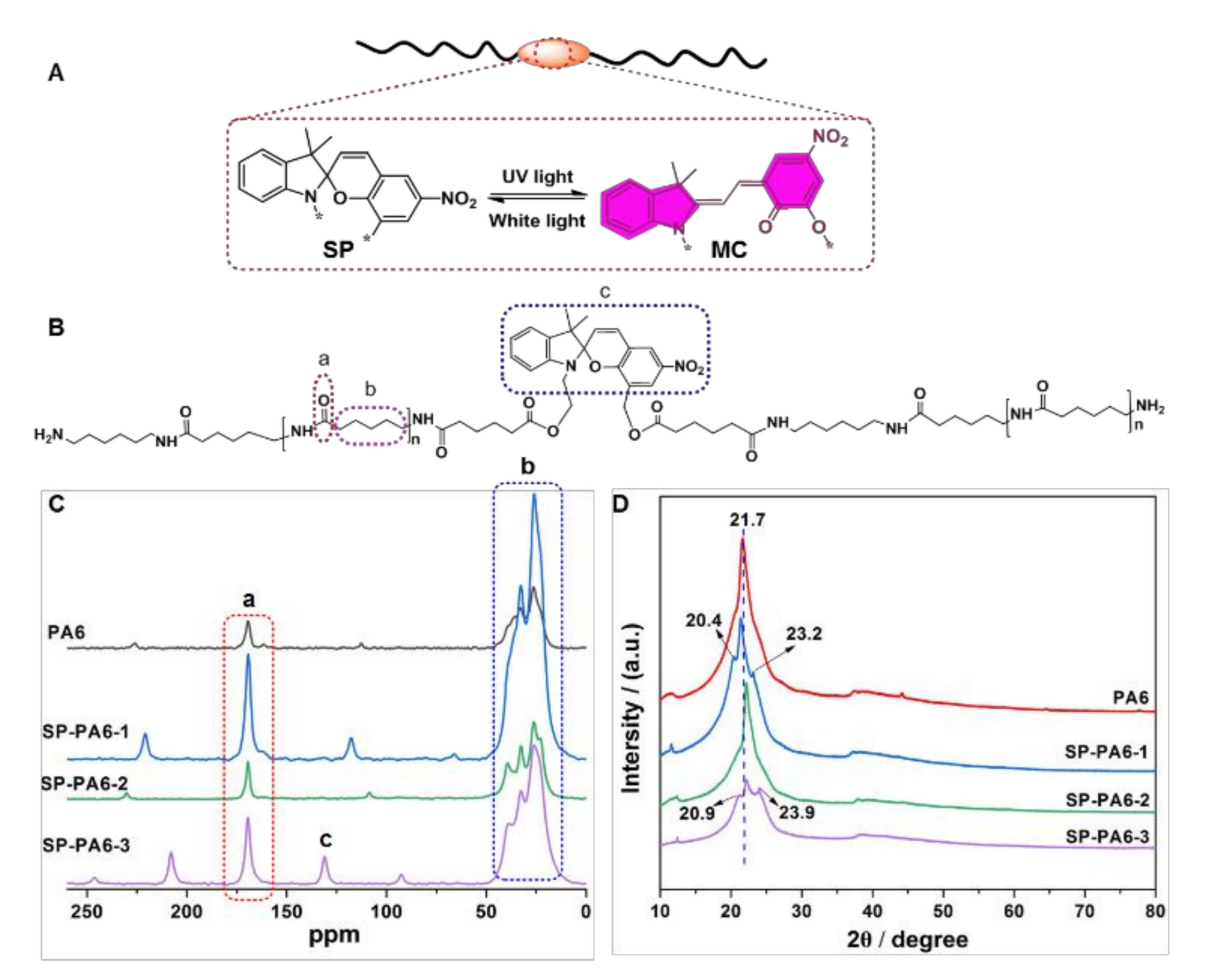
(**A**) The scheme of photochromic PA6 and photochromic mechanism. (**B**) The chemical formula of SP-modified PA6. (**C**) ^13^C NMR spectra and (**D**) XRD profiles of PA6 and SP-modified PA6 samples.

The photochromic mechanism is shown in Figure 3A. From Figure 3A, it can be seen that spiropyran features reversible transformation between two forms, namely, the colorless ring-closed SP form and the colored ring-opened merocyanine (MC) form upon UV light stimuli [13]. Figure 3B shows the chemical structure of SP-modified PA6 and the ^13^C NMR spectra are shown in Figure 3C. The ^13^C NMR spectrum of pure PA6 is also shown for comparison. The chemical shifts of carbon atoms in carbonyl and alkyl groups can be seen in all spectra at 169 ppm and 25–39 ppm, respectively. Moreover, the chemical shift of carbon atoms in benzyl groups of spiropyran can be seen in ^13^C NMR spectrum of SP-PA6-3, which certify that spiropyran can be chemically bonded into the polymer chains of PA6.

X-ray diffraction (XRD) was carried out to characterize the microstructure of PA6 and SP-modified PA6 (Figure 3D). PA6 and SP-PA6-2 are thoroughly crystallized in the γ-form (2θ ≈ 21.7°) according to Arimoto’s unit cell [36]. In contrast to pure PA6, the curve of SP-PA6-1 reveal the two scattering peaks at 2θ ≈ 20.4° and 2θ ≈ 23.2° associated with the α (200) and α (002/202) planes [37]. SP-PA6-3 show a similar crystallization behavior with two scattering peaks at 2θ ≈ 20.9° and 2θ ≈ 23.9°. However, broad scattering peak exists in the curve of SP-PA6-3, which is attributed to that the incorporation of more HOOC-SP-COOH into PA6 chains influenced the crystallization behavior of the resulting copolymers and decreased crystallinity degree since only the segments of PA6 can crystallize.

**Figure 4:**
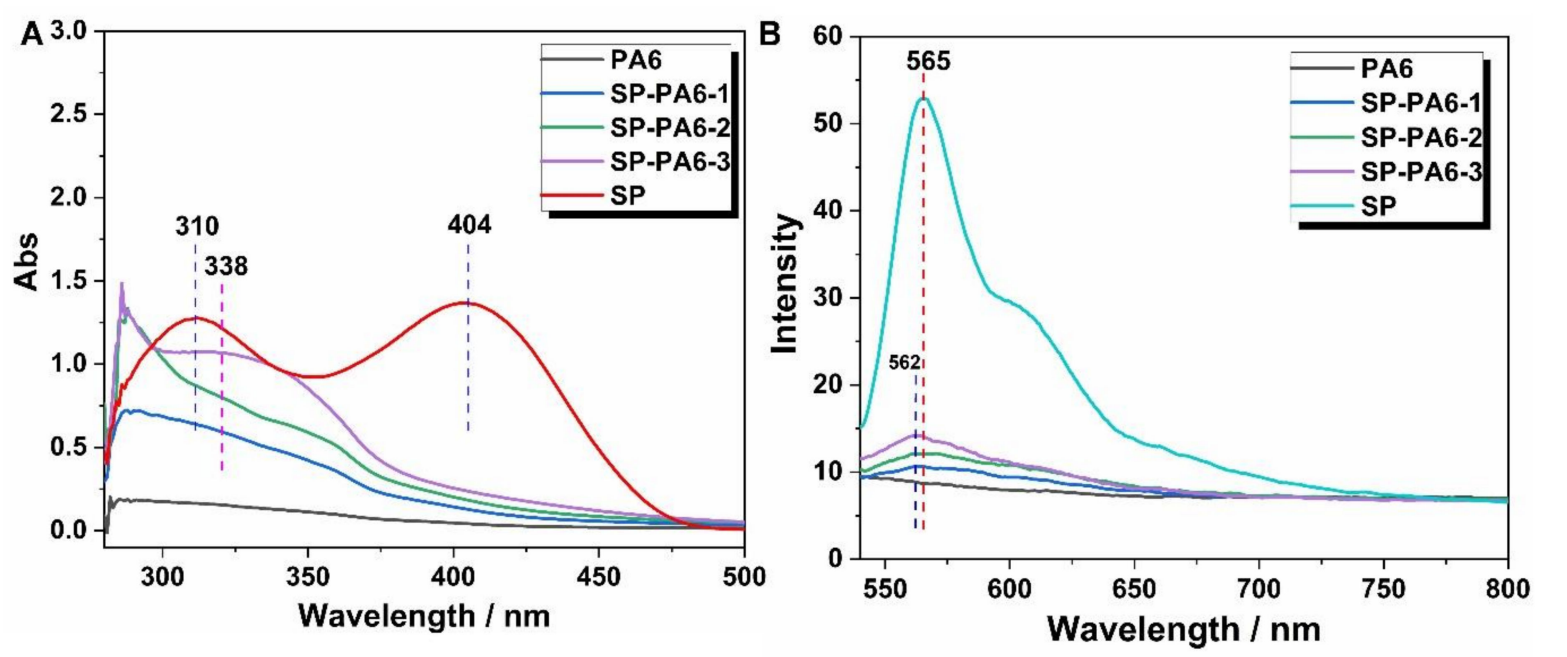
(**A**) UV-visible absorbance spectra and (**B**) fluorescence spectra of PA6 and SP-modified PA6 in formic acid (0.03 g/mL) at 298 K (λ_ex_ = 523 nm).

To further demonstrate the structure of SP-modified PA6, UV-visible absorbance spectra and fluorescence spectra were measured, which are displayed in Figure 4. UV-visible absorbance spectrum of spiropyran/HOOCH solution in Figure 4A reveals that absorbance peaks at 310 nm and 404 nm are assigned to the characteristic peaks of spiropyran and MCH [8,26]. From spectra of SP-modified PA6, it can be seen that absorbance peak of spiropyran occur in all samples and the peak of SP-PA6-3 sample are strongest at the same concentration which proves the high content of spiropyran derivative in PA6 polymer chains. However, the absorption peak of spiropyran in SP-PA6-3 shifted to 338 nm. In addition, peak at 565 nm emerged in the fluorescence emission spectra (λ_ex_ = 523 nm) in Figure 4B, which is assigned to the ring-closing isomer of spiropyran. It also can be seen from Figure 4B that the fluorescence intensity increases from SP-PA6-1 to SP-PA6-3 with increasing the content of the spiropyran derivative. In addition, the peaks shifted a little to 562 nm.

### 4.3. Thermal and Mechanical Properties

**Figure 5:**
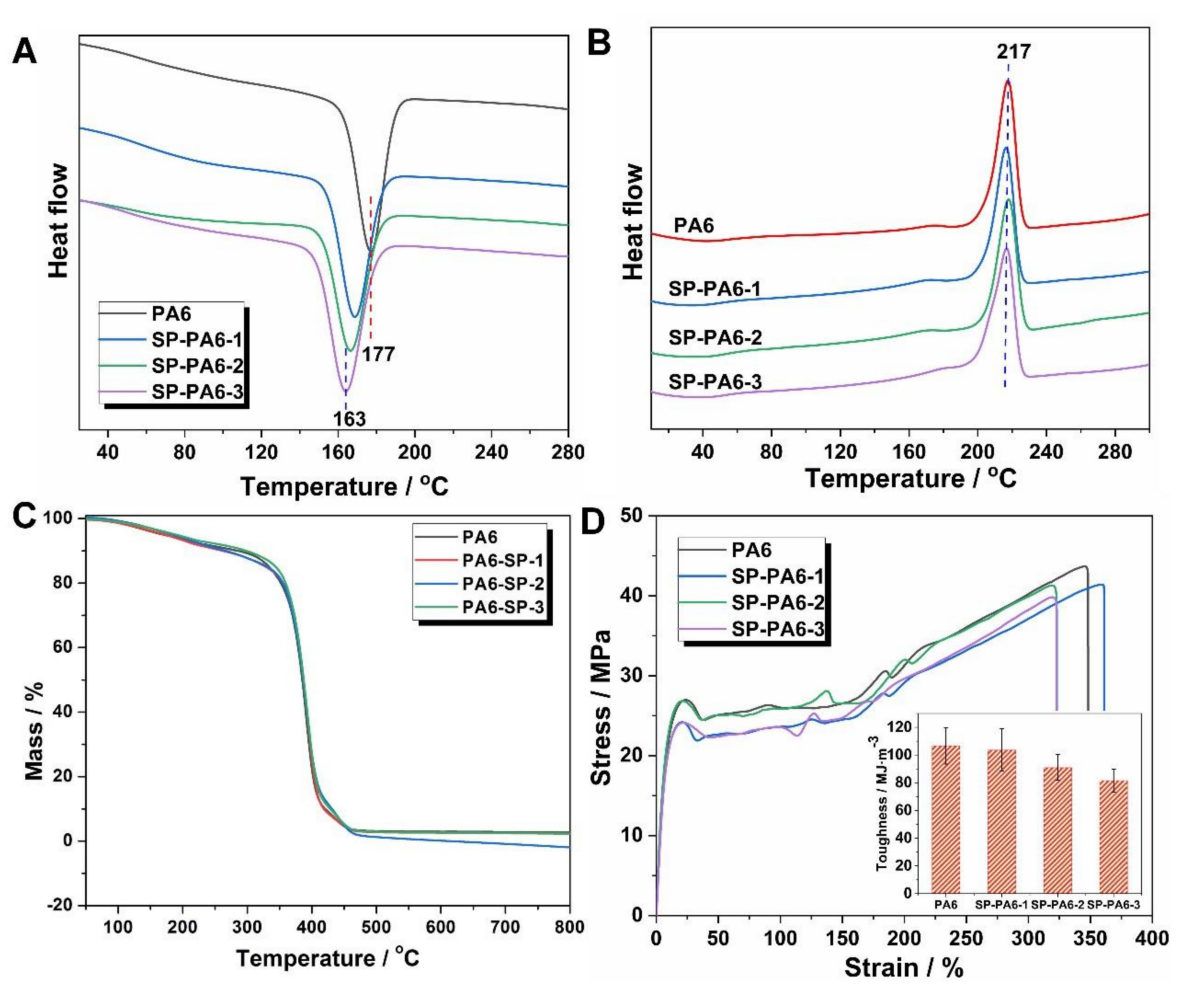
DSC thermograms for PA6 and SP-modified PA6, (**A**) heating ramp and (**B**) cooling (crystallization) ramp. (**C**) Thermogravimetric analysis traces of PA6 and SP-modified PA6. (**D**) Typical stress-strain curves and toughness of PA6 and SP-modified PA6.

Figure 5 shows the DSC crystallization curve and melting curve for pure PA6 and SP-modified PA6. Melting temperature (*T*_m_) and crystallization temperature (*T*_c_) of all samples were determined from the curves, which are also been shown in Table 2. As we know, *T*_c_ of PA6 has a strong dependence on their chemical structure [37]. In Figure 5A, *T*_c_ of SP-PA6-3 is the lowest, which is attributed to the chain flexibility increases because of the high content of spiropyran derivative. Furthermore, *T*_c_ departure of different samples may be connected with their crystal structure habits in relation to the chemical structures and nucleation process. It also exhibits from Figure 5B that *T*_m_ of SP-PA6-1 to SP-PA6-3 do not vary a lot comparing to that of pure PA6. The thermogravimetric analysis traces in Figure 5C show that the thermal degradation rate of SP-modified PA6 is the largest at approximate 380 °C. It can be observed from the mechanical properties in Figure 5D and Table 2 that toughness of photochromic PA6 was decreased when SP derivative was chemically embedded into PA6 chains with result in decreasing chain regularity and crystallinity degree. It is with accordance to the result of XRD profiles.

### 4.4. Photochromic Properties of SP-Modified PA6

**Figure 6:**
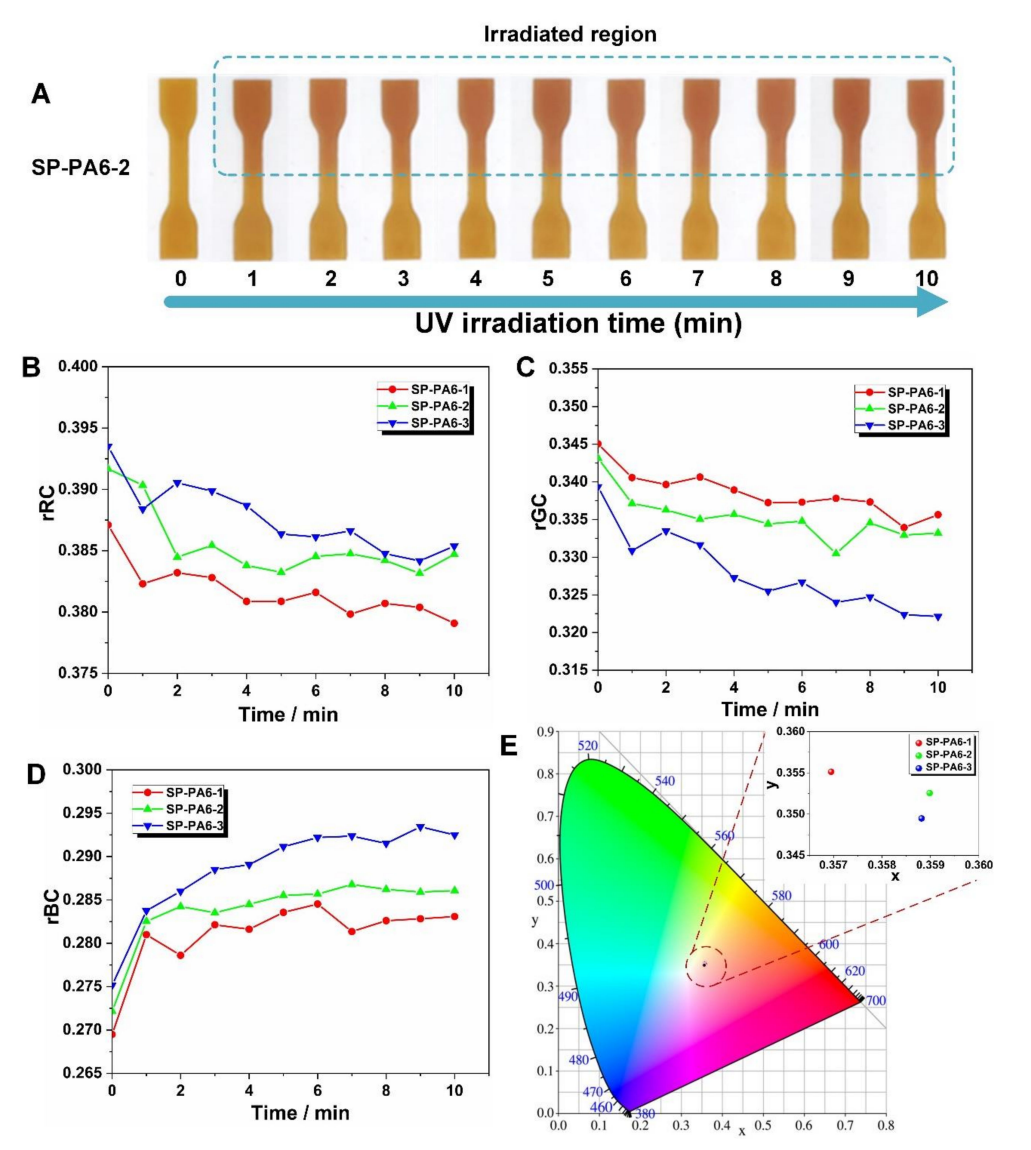
(**A**) Photographs of SP-PA6-2 after UV irradiation for different times. (**B**) rRC (red), (**C**) rGC (green) and (**D**) rBC (blue) signals as a function of irradiation time for different test samples. (**E**) The CIE 1931 chromaticity diagram of SP-PA6-1, SP-PA6-2 and SP-PA6-3 after being irradiated for 1 min.

The photochromic properties of SP-modified PA6 were investigated by monitoring the photoresponse characteristic under UV irradiation (λ = 365 nm). In Figure 6A and Figure S2, all the samples possess the sensitively photochromic phenomenon. For quantitative analysis of color properties, ImageJ software was used to determine the color characteristic values (RGB) of different test samples (Figure 6B,D). The color change of the photochromic PA6 after being irradiated for 1 min is measured by CIE 1931 (International Commission on illumination) color space (Figure 6E). From the calculation of the RGB ratio and the result in CIE 1931 chromaticity diagram, we found that the sample SP-PA6-3 was more sensitive to UV light at the beginning of irradiation than other samples (Figure 6E). In addition, its color after being irradiated for 1 min tended to reddish. The highly sensitive photoresponse is ascribed to the polymer chains with more flexibility and more content of SP.

### 4.5. Photoresponse Reversibility and Fatigue Resistance

**Figure 7:**
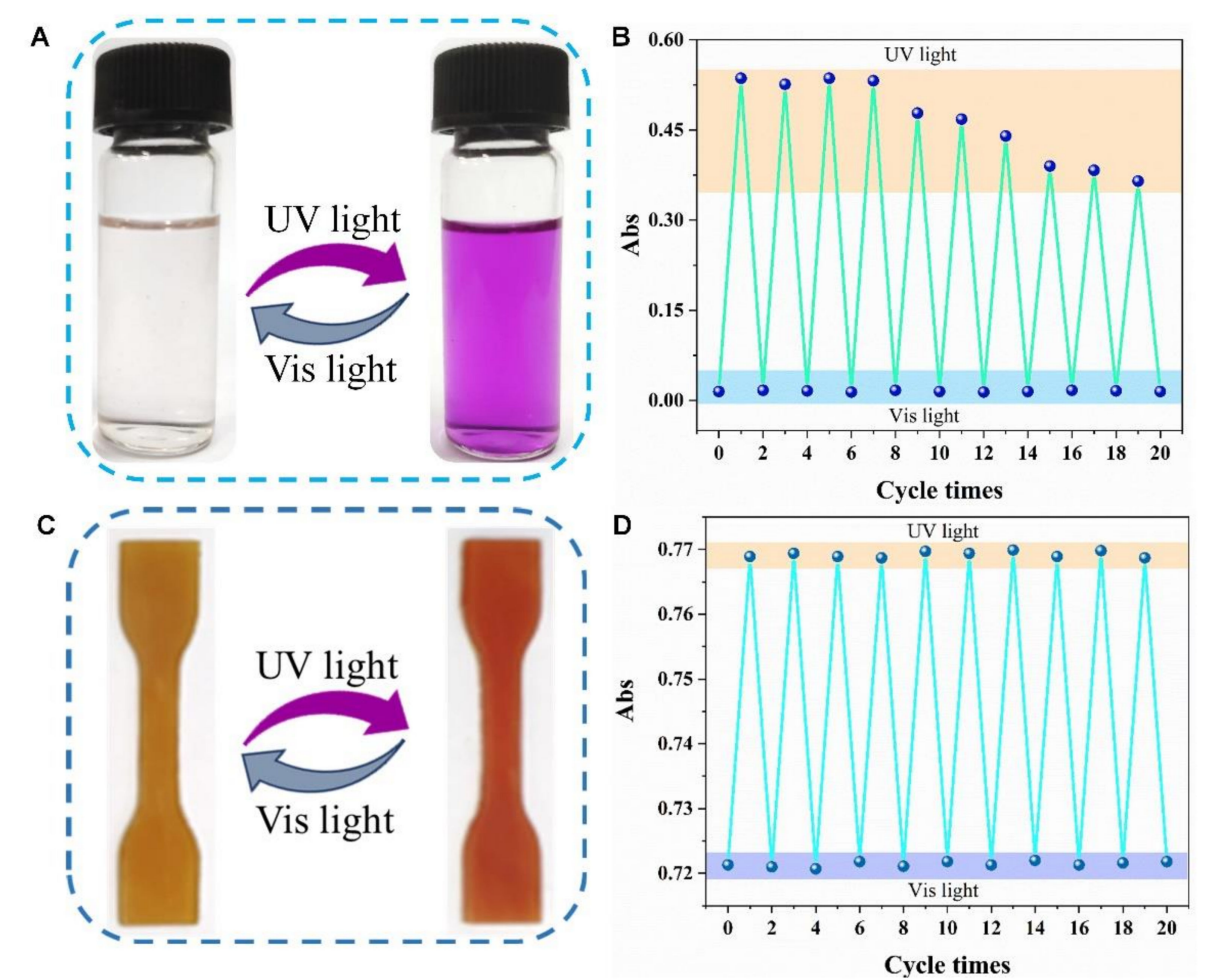
(**A**) The photograph for photoresponse reversibility of HOOC-SP-COOH/ethanol solution, (**B**) the fatigue resistance of HOOC-SP-COOH/ethanol solution, (**C**) the photograph for photoresponse reversibility of SP-PA6-3 and (**D**) the fatigue resistance of SP-PA6-3.

Photoresponse reversibility and fatigue resistance are important in photochromic materials, which determine the service life. Photoresponse reversibility of HOOC-SP-COOH/ethanol solution upon alternating cycles of UV (365 nm, 60 s) and visible (60 s) irradiations was investigated firstly, which is shown in Figure 7A,B and absorbance intensity variations of HOOC-SP-COOH/ethanol solution in the UV-visible absorbance spectra at λ_max_ = 542 nm were recorded upon the aforementioned stimuli during 10 cycles (Figure S3). It can be seen that the photoresponse of HOOC-SP-COOH/ethanol solution was reversible but the absorbance of HOOC-SP-COOH/ethanol solution decreased by 31.9% after 10 cycles.

Additionally, photoresponse reversibility and fatigue resistance of SP-modified PA6 were also studied, which are shown in Figure 7C,D. In Figure 7C, with comparison to HOOC-SP-COOH/ethanol solution, the color change of SP-modified PA6 under UV irradiation is weak, which is attributed to the steric effect in crystallized polymer chains of PA6 to limit the isomerization of SP-MC. In Figure 7D, the process was carried out to investigate the stability and switchability between SP and MC upon UV-vis irradiation cycles (UV irradiation for 10 min and then visible light irradiation for 6 min) and also photostability and photoreversibility of MC. Variation in the absorbance intensity of SP-PA6-3 at λ_max_ = 575 nm was recorded during 10 cycles (Figure S4). It reveals that the absorbance of SP-PA6-3 had negligible decay after 10 cycles, which indicates the SP-modified PA6 possessed excellent photoresponse reversibility and fatigue resistance. As we know, it is the first report for photochromic polyamide material with SP as molecular component. Moreover, the present results display that photoresponse fatigue resistance of SP-modified PA6 is better than spiropyran-containing fluorinated polyacrylate [18], spiropyran enantiomeric glutamate gels [24] and photochromic cotton fabric based on microcapsule coating [38].

## 5. Conclusions

In this study, spiropyran diol was synthesized and then it reacted with adipic acid to obtain the modifier (HOOC-SP-COOH). Amido-terminated polyamide 6 was synthesized by hydrolyzed ring-opening polymerization of ε-caprolactam and hexamethylenediamine. Amido-terminated polyamide 6 was modified by HOOC-SP-COOH for photochromic PA6. ^13^C NMR spectra characterization of the chemical structures revealed that spiropyran could be chemically bonded into the chains of PA6. With increasing content of HOOC-SP-COOH, crystallinity degree of the resulting copolymers was decreased since only PA6 segments can crystallize. Meanwhile, toughness of photochromic PA6 was decreased. The photochromic property analysis indicated that the sample SP-PA6-3 was more sensitive to UV light at the beginning of irradiation than other samples and its color after being irradiated for 1 min tended to reddish, which was ascribed to the polymer chains with more flexibility and more content of SP. Investigation revealed that the UV-vis absorbance of SP-PA6-3 had negligible decay after 10 cycles, which indicated SP-modified PA6 possessed excellent photoresponse reversibility and fatigue resistance. The photochromic PA6 synthesized in this work has potential application in UV sensors and textile materials, etc.

## Figures and Tables

**Table 1:** Synthesis formulation of photochromic PA6.

Samples	CPL(g)	H_3_PO_4_(g)	H_2_O(g)	HDA(g)	HOOC-SP-COOH			
					SP Diol (mg)	Adipic Acid (mg)	DCC (mg)	DMAP (mg)
PA6	50.012	1.002	0.503	0	0	0	0	0
SP-PA6-1	50.002	1.002	0.501	0.25	25.0	47.45	26.9	7.9
SP-PA6-2	50.003	1.001	0.501	0.25	30.0	57.67	32.3	9.6
SP-PA6-3	50.004	1.001	0.500	0.25	40.0	76.65	43.3	12.3

**Table 2:** Characteristics of pure PA6 and photochromic PA6.

Samples	*M*_n_^a^ (g/mol)	*M*_w_^a^ (g/mol)	*M*_w_/*M*_n_^a^	*T*_c_^b^ (°C)	*T*_m_^b^ (°C)	Toughness ^c^ (MJ/m^3^)
PA6	16,200	26,200	1.62	177	217	106.6 ± 13.2
SP-PA6-1	17,600	23,700	1.35	168	216	103.7 ± 15.2
SP-PA6-2	18,900	28,300	1.50	166	218	91.0 ± 9.3
SP-PA6-3	31,100	58,400	1.87	163	217	81.6 ± 8.2

^a^ Measured by Gel Permeation Chromatography (GPC). ^b^ Measured by DSC curves. ^c^ Measured by tensile test.

## Data Availability

The data presented in this study are available on request from the corresponding author.

## Supplementary Materials

The following are available online at https://www.mdpi.com/article/10.3390/polym13152496/s1, Figure S1: Spectrum of HOOC-SP-COOH measured by LC-MS. Figure S2: Photos of SP-PA6-1 and SP-PA6-3 after being irradiated for different time. Figure S3: UV-vis adsorption spectra of HOOC-SP-COOH/ethanol, Figure S4 UV-vis adsorption spectra of SP-PA6-3.
