# Supplementary material for: Photochromic Polyamide 6 Based on Spiropyran Synthesized via Hydrolyzed Ring-Opening Polymerization
Source: Polymers (Basel). 2021 Jul 28;13(15):2496. doi: 10.3390/polym13152496 (PMC8348056; doi:10.3390/polym13152496)
Supplement: Supplementary file 1 [file polymers-13-02496-s001.zip › polymers-1308764-supplementary.pdf]

Supporting information

Photochromic polyamide 6 based on spiropyran synthesized via hydrolyzed ring-opening polymerization

Shiyou Tian,[1, 2, #] Jicong Zhang,[1, 2, #] Qiong Zhou,[3] Limei Shi,[3] Wenwen Wang[1,2, *],
Dong Wang[1,2]

[1] Key Laboratory of Textile Fiber and Products (Wuhan Textile University), Ministry of Education, Wuhan 430200, China;

[2] Hubei International Scientific and Technological Cooperation Base of Intelligent Textile Materials & Application, Wuhan Textile University, Wuhan 430200, China

[3] SINOPEC Yizheng Chemical Fiber Co., Ltd., Jiangsu Key Laboratory of Highperformance Fiber, Yizheng 211900, China

Liquid Chromatograph-Mass Spectrometry (LC-MS).

The chemical formula of HOOC-SP-COOH is $C_{33}H_{38}N_2O_{11}$, and the theoretical m/z is 638.25. From **Figure S1**, it can be seen that the peak at 639 ($(M+H)^+$, m/z) is the strongest, which shows the high purity of SP derivative.

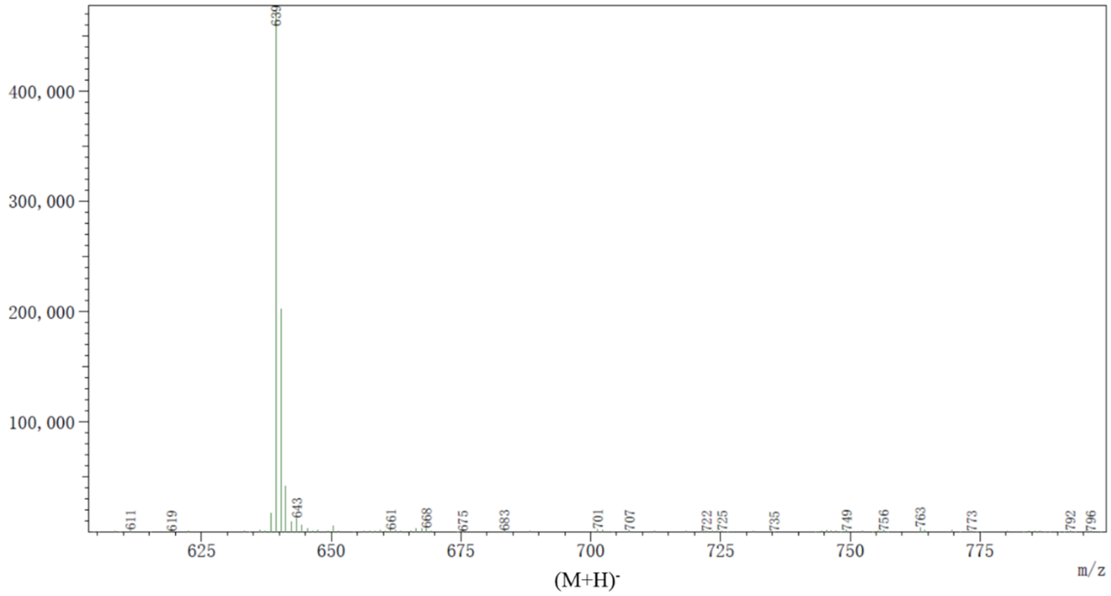

Figure S1 Spectrum of HOOC-SP-COOH measured by LC-MS.

Photochromic property of SP-PA6-1 and SP-PA6-3.

[#]The authors contribute equally to this work.
[*]Corresponding author. Email: wwang@wtu.edu.cn.

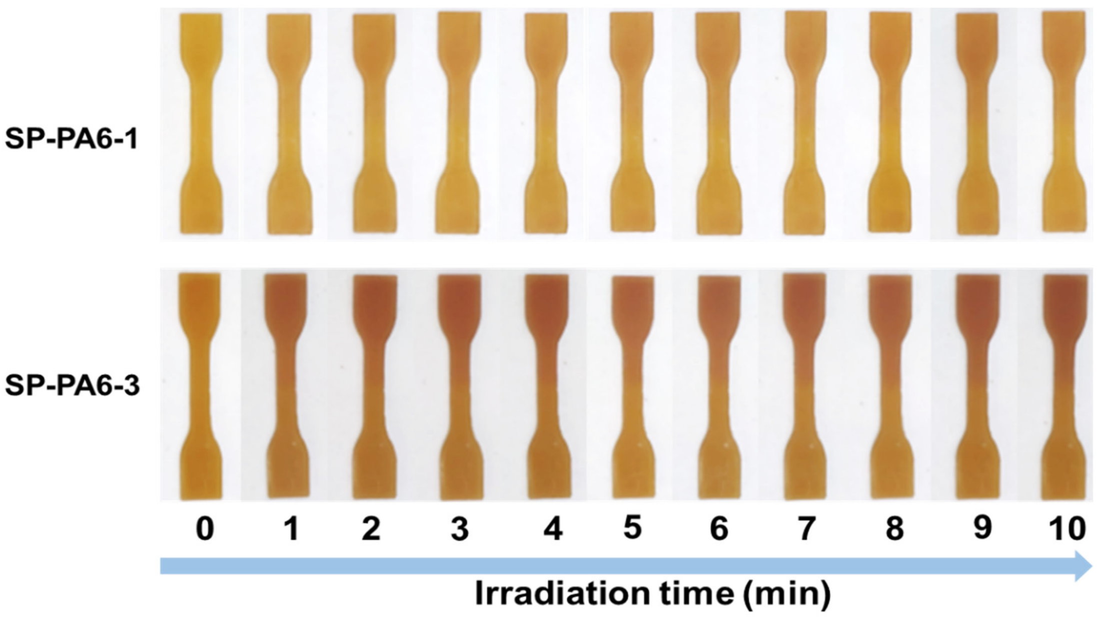

Figure S2 Photos of SP-PA6-1 and SP-PA6-3 after being irradiated for different time.

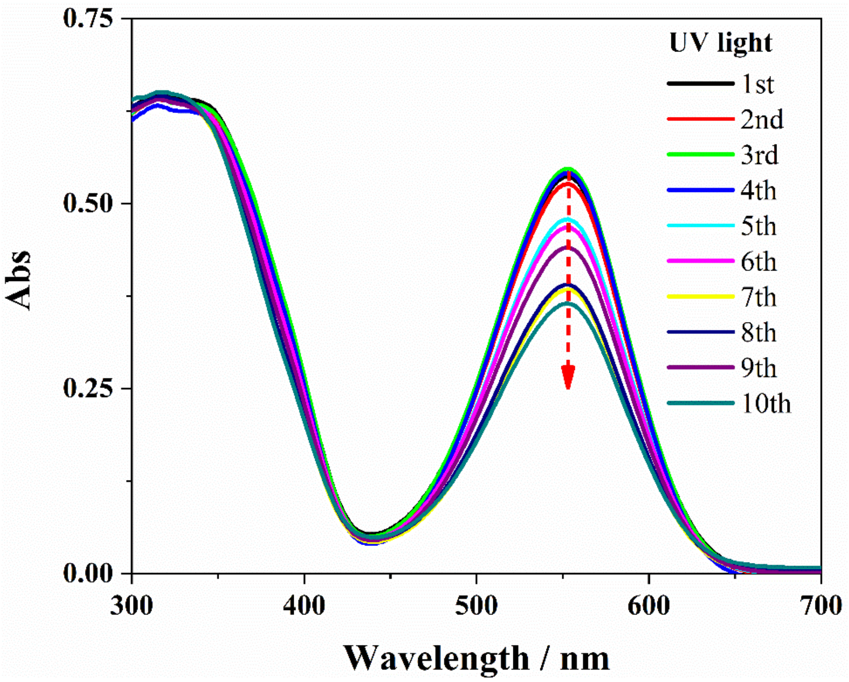

Figure S3 UV-vis adsorption spectra of HOOC-SP-COOH/ethanol

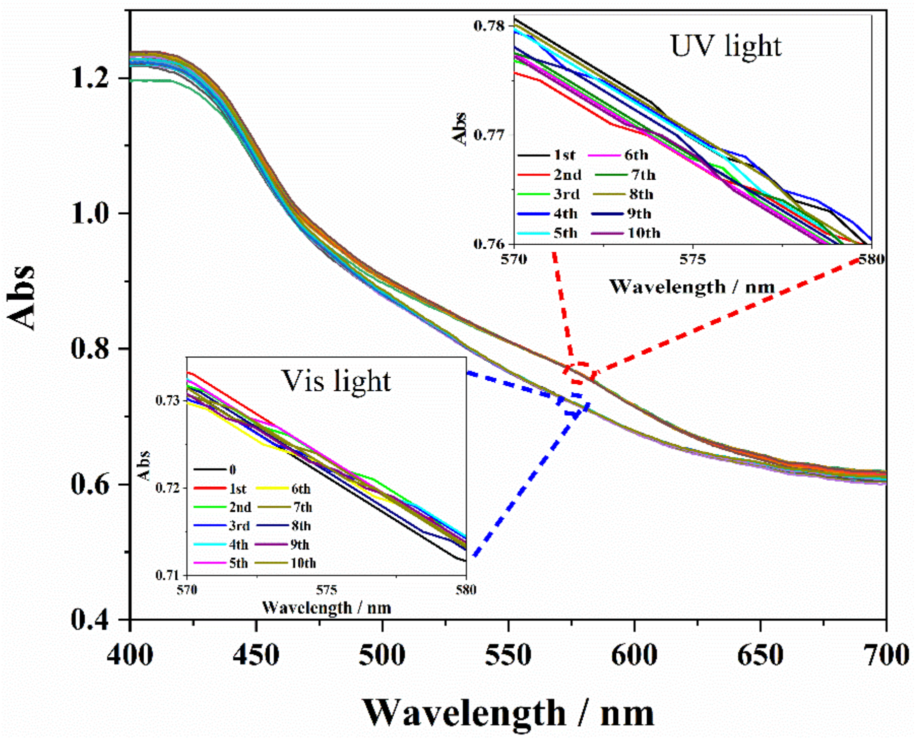

Figure S4 UV-vis adsorption spectra of SP-PA6-3
